# Supplementary material for: Interconnected Set of Enzymes Provide Lysine Biosynthetic Intermediates and Ornithine Derivatives as Key Precursors for the Biosynthesis of Bioactive Secondary Metabolites
Source: Antibiotics (Basel). 2023 Jan 12;12(1):159. doi: 10.3390/antibiotics12010159 (PMC9854754; doi:10.3390/antibiotics12010159)
Supplement: Supplementary file 1 [file antibiotics-12-00159-s001.zip › antibiotics-2132669-supplementary.pdf]

Table S1. Enzymes and genes involved in the biosynthesis of pipecolic acid*

**Enzyme**	**Reaction**	**Organism**	**Gene**
Homocitrate synthase	a-Ketoglutarate + Acetyl-CoA →Homocitrate	Fungi	lys1
Homoaconitase	Homocitrate → Homoisocitrate	Fungi	*lys3/lysF*
Homoisocitrate Dehydrogenase	Homoisocitrate → a-Ketoadipate	Fungi	*lys4*
a-Ketoadipate aminotransferase	a-Ketoadipate → a-Aminoadipic acid	Fungi	*lys6*
a-Aminoadipate reductase	a-Aminoadipic acid → a-Aminoadipate semialdehyde	Fungi	*lys2*
PPTse	a-Aminoadipic acid → a-Aminoadipate semialdehyde	Fungi	*lys5*
Pipecolate oxidoreductase	a-Aminoadipate semialdehyde → Pipecolic acid	Fungi	---
Saccharopine Dehydrogenase	Saccharopine → Lysine<br>Lysine → Saccharopine	Fungi	*lys8*
Saccharopine Reductase	Saccharopine → a-Aminoadipate semialdehyde<br>a-Aminoadipate semialdehyde → Saccharopine	Fungi	*lys7*
Saccharopine Oxidase	Saccharopine → a-Aminoadipate semialdehyde	Fungi	*fap2*
Lysine w aminotransferase	Lysine → a-Aminoadipate semialdehyde	Fungi	w-at
Lysine cyclodeaminase	Lysine → Pipecolic acid	Bacteria	*rapL*<br>*fkbL*
Response defense aminotransferase	lysine → 2,3-dehydropipecolic	Plants	*ald1*
Systemic adquired resistance reductase	2,3-dehydropipecolic → Pipecolic acid	Plants	*sard4*

- Notice that some enzymes have been studied but the genes have not been cloned.
- The names of the genes corresponds to filamentous fungi and are not the same than in yeasts

Table S2. Enzymes and Genes involved in the biosynthesis of 4-Oxopipecolic acid and 3-Hydroxypicolinic acid

Enzymes	Reaction	Organisms	Gene
Lysine cyclodeaminase	Lysine Pipecolic acid	Bacteria	*pipA*
Pipecolate 4 monooxygenase	Pipecolic 4 Oxopipecolic	Bacteria	*snbF, visD*
Lysine-2-aminoatransferase	Lysine →2-keto, 6 aminocaproic acid (P2C)	Bacteria	*hpaA, visA*
2 Keto, 6 aminocaproic acid cyclase*	2-keto, 6 aminocaproic acid→ 3 Hydroxypicolinic acid	Bacteria	-----

*The enzyme converting 2-keto, 6 aminocaproic acid→ 3 Hydroxypicolinic acid has not been fully characterized
